# Acute effect of kinesio tape on postural control in individuals with functional ankle instability following ankle muscle fatigue

**DOI:** 10.3389/fphys.2022.980438

**Published:** 2022-08-30

**Authors:** Pan Li, Zhen Wei, Ziwei Zeng, Lin Wang

**Affiliations:** Key Laboratory of Exercise and Health Sciences, Shanghai University of Sport, Ministry of Education, Shanghai, China

**Keywords:** functional ankle instability, kinesio tape, fatigue, postural control, short term

## Abstract

**Background:** Kinesio taping (KT) is one of the therapeutic interventions in sports medicine practice. The study aims to assess the acute effect of different KT methods on postural control in individuals with functional ankle instability (FAI) after ankle muscle fatigue.

**Methods:** Twenty-eight participants with FAI were recruited to complete maximum voluntary isometric contraction (MVIC) and proprioception of ankle using isokinetic dynamometer, dynamic postural control using Y-balance test and static postural control using a force platform after a fatigue protocol in four taping conditions: facilitatory KT (FKT), ankle balance taping (ABT), sham taping (ST) and no taping (NT).

**Results:** No significant difference was observed for the data MVIC and proprioception after ankle muscle fatigue amongst the four taping treatments. A significant difference in Y-Balance Test was observed amongst the four taping treatments at posterolateral direction (*p* < 0.001) and posteromedial direction (*p* < 0.001), suggesting that KT may significantly improve dynamic postural control following ankle muscle fatigue. For Center of pressure (COP) measurements, the mediolateral COP sway range of NT was significantly larger than that of FKT (*p* = 0.003) and ST (*p* < 0.001), suggesting that the placebo effect of KT was inevitable.

**Conclusion:** The effect of KT seems increased dynamic postural control in individuals with FAI after ankle muscle fatigue, and this effect is not strongly related to the taping methods. By preventing fatigue-related impairments of postural control, KT may help reduce the risk of injury in individuals with FAI.

## Introduction

Lateral ankle sprain has a high occurrence, not only in physically active patients but also in the general populations (Gribble et al., 2016). Despite the high prevalence, people rarely seek interventions after a first sprain (Feger et al., 2017). The high rate of reinjury leads to chronic ankle instability (CAI), characterised by persistent ankle pain, swelling, self-reported instability and feelings of “giving way” (Gribble et al., 2014). Repeated ankle sprains increase the physical and mental burden on patients. Hiller et al. (2011) proposed a model of CAI involving mechanical ankle instability, functional ankle instability and recurrent ankle sprain independently or in combination. Functional ankle instability (FAI) refers to the tendency of the ankle to sprain or the feeling of “giving way” after an initial ankle sprain.

Previous research has indicated that FAI is associated with neuromuscular deficit, including a decrease in muscle strength, proprioception and postural control (Hertel, 2002). In addition, most ankle injuries occur during the last third of each half of matches, where athletes have insufficient rest (Woods et al., 2003). This phenomenon may indicate that ankle sprain may be related to fatigue state. Fatigue is defined as the inability to maintain the required or expected strength (Edwards, 1981). It is a major factor increasing the risk of falls and acute injury which has been well documented (Paquette et al., 2017). Possible contributions of fatigue in performance have been attributed to decreases in postural control and proprioception (Verschueren et al., 2020). In addition, compared with healthy people, fatigue is more harmful to people with FAI (Jahjah et al., 2018). Therefore, it is very important to potentially decrease fatigue in sport.

Kinesio taping (KT) is one of the therapeutic interventions in sports medicine practice. Previous studies have attempted to explain the benefits of KT on muscle strength, range of motion, proprioception, functional performance, pain and postural control (Binaei et al., 2021; Biz et al., 2022). KT is also used to prevent ankle sprain and as an intervention to improve function in individuals with CAI (Sarvestan et al., 2020). However, the results of many studies evaluating the efficacy of KT for CAI are controversial. In a systematic review, Wang et al. (2018) concluded that KT was superior to other taping methods in ankle functional performance and it may be useful to athletes with ankle sprains. In another systematic review published in the same journal, Nunes et al. (2021) did not obtain sufficient evidence to encourage using KT for functional performance in people with or without ankle sprain. One of the main reasons for these conflict results may be the huge differences in recruited participants. The first review included only 10 studies (233 participants), while the second included 84 studies (2684 participants) and discussed the different populations separately. Another reason for these conflict results could be the inconsistent techniques of KT (Lim and Tay, 2015). Therefore, it is difficult to draw a definite conclusion for CAI individuals.

In relation to the application of KT on the ankle joint, some studies have used facilitatory KT (FKT) (Halseth et al., 2004) and some have used ankle balance taping (ABT) (Yin et al., 2021), but few studies have compared the effects of the two taping methods. In addition, a dearth of determining the possible benefits of KT on postural control in people with FAI after muscle fatigue still exists. Thus, this study aimed to assess the acute effect of different KT methods on postural control in individuals with FAI after ankle muscle fatigue.

## Materials and methods

### Participants

The G-Power was used to perform a priori power analysis. Considering the effective size of 0.25 (Cohen, 1988), the power of 0.8, and alpha level of 0.05, twenty-eight participants (15 males, 13 females; age, 21.2 ± 2.0 years; body height, 172.3 ± 8.0 cm; body weight, 64.1 ± 10.1 kg) were recruited in this study. The inclusion criteria (Wu et al., 2022) are as follows: 1) a history of at least one significant ankle sprain (initial must have occurred at least 1 year prior to study enrolment); 2) feelings of “giving way” of the ankle joint, and/or recurrent sprain, and/or “feeling of instability”; 3) a score of Identification of Functional Ankle Instability of at least 11 (Simon et al., 2012) (if both feet match, the less stable side is chosen); 4) negative anterior drawer test and talar tilt test; and 5) not allergic to KT. The participants were free of lower extremity musculoskeletal surgeries, operation, nervous and vestibular system disease or any other conditions that could affect to postural control. Those who had an acute ankle sprain within 3 months prior to the study, resulting in cessation of physical activity for at least 1 day, were excluded (Yin et al., 2021). All participants were asked to provide consent before the experiments. This study was approved by the Human Ethics Committee of the Shanghai University of Sport.

### Taping methods

**FIGURE 1:**
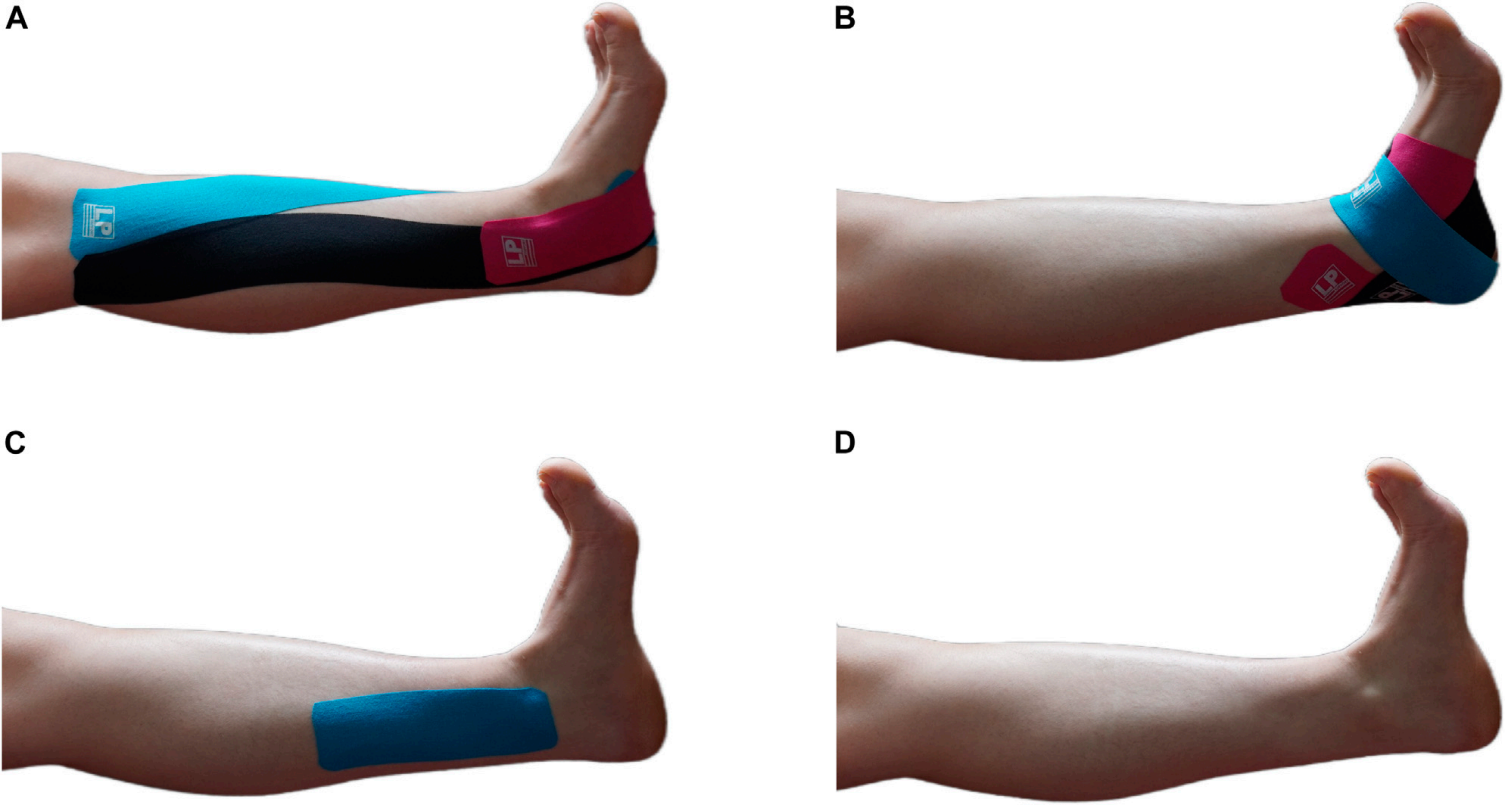
Four treatments in this study. **(A)** FKT, facilitatory Kinesio taping; **(B)** ABT, ankle balance taping; **(C)** ST, sham taping; **(D)** NT, no taping.

Before taping, the skin of taping area was shaved and wiped with 70% alcohol (Saltan et al., 2019). Each participant randomly experienced four taping conditions as follows: 1) FKT (Halseth et al., 2004); 2) ABT (Lee and Lee, 2015); 3) sham taping, (ST) (Lee and Lee, 2015), and 4), no taping (NT, Figure 1). The taping (LP 670 MAXTAPING) was applied before the fatigue protocol. Additionally, a wash-out phase of 1 week was performed between each taping treatment to relieve any learning effect.

All tapes were accomplished by a single experienced physical therapist who was not involved in the recruitment and assessment. The FKT and ABT consistency with 50% tension was maintained using the following equation (Wei et al., 2020), and the ST was applied without tension.

$$\text{Actual length of tape to cut }\left(\text{cm}\right)\;=\;\left[\frac{x-4}{1.5}+4\right]\;\times \;1.1$$

where x is the actual length measured from the origin and insertion and the anchor length of the KT was set to 4 cm (2 cm each for the proximal and distal sites).

### Fatigue protocol and maximum voluntary isometric contraction measurement

The fatigue protocol was performed on a CONTREX isokinetic dynamometer (PHYSIOMED CON-TREX TP1000, Germany) in accordance with previous studies (Gribble et al., 2007). The participants were instructed to avoid muscle fatigue 48 h prior to assessment. They were secured in the isokinetic device with their ankle at neutral position and they were stabilised with two straps around the abdomen. The movement pattern was set at concentric/concentric mode and the angular velocity at 60°/s. Prior to fatigue protocol, the participants performed 10 repetitions of consecutive maximal concentric/concentric contractions of the ankle to familiarise the device. Then, they rested for 5 min to restore muscle strength. The maximum voluntary isometric contraction (MVIC) of plantarflexion and dorsiflexion for 5 s was measured three times by a rest period of 30 s before inducing the ankle muscle fatigue. The highest value of the MVIC was taken as a parameter for inducing fatigue. Next, each participant was asked to perform plantarflexion and dorsiflexion contractions at maximal effort. Verbal encouragement was given by the examiner. According to previous studies (Flevas et al., 2017), the point of muscle fatigue must meet the following two criteria: 1) three consecutives of the peak torque reaching below 50% MVIC of the first measured and 2) the participants reported they were no longer able to complete any repetitions. After the fatigue protocol was conducted, three times of 5 s of MVIC of plantarflexion and dorsiflexion by a rest period of 30 s were performed once again and used in data analysis.

### Joint position sense measurement

Ankle proprioception is an important component of postural control in movement because in most physical activities, the ankle-foot complex is the only part of the body that touches the ground (Han et al., 2015). The ankle proprioceptive tests in the current study included Joint position sense (JPS) and force sense (FS) measurements. JPS was assessed using CONTREX and tested at 5° of plantarflexion and 5° of dorsiflexion. Firstly, the participant’s ankle was passively moved from the neutral position by the isokinetic force measurement system to one of two target positions randomly and stayed for 10 s. The participants were then asked to concentrate on the feeling of position of this angle and then took the initiative to acquire the angle of practice twice. Before the test, the participants were instructed to avoid active muscle contraction and blindfolded, with ears covered throughout the examination. The participants held the hand-held switch and actively started to move from the neutral position of the ankle joint. When the participants felt that the ankle joint reached the target angle, the switch was pressed and the tester recorded the actual angle. This experiment was repeated in three trials, and the experimental results of each trial were not fed back to the participants.

The accuracy of JPS was inversely proportional to the absolute error (AE) and variable error (VE) scores. AE is a measure of the overall accuracy of the positioning and VE is a measure of the variability of the positioning (Jahjah et al., 2018). The formulas are as follows:

$$\text{AE}=\frac{\sum_{\text{i}=1}^{3}\left|{\text{a}}_{\text{i}}-\text{a}\right|}{3}\;$$ (1)

$$\text{VE}=\sqrt{\frac{\sum_{\text{i}=1}^{3}{\text{a}}_{\text{i}}^{2}-\frac{{\left(\sum_{\text{i}=1}^{3}{\text{a}}_{\text{i}}\right)}^{2}}{3}}{2}}$$ (2)

The actual measured value is *a*_*i*_ and the target value is *a*.

### Force sense measurement

All participants were placed in the same position as in JPS measurement. The main purpose was to examine the ability of the participant’s ankle joint to replicate the torque value of 25% MVIC. Before the force sense test was performed, the participants were asked to plantarflex/dorsiflex to a torque of 25% MVIC, with looking at a monitor showing the torque, and keep the position for 10 s. The participants had to concentrate on the feeling of target force and then withdraw from the monitor. The participants were asked to relax and close their eyes. Next, they were asked to reproduce that target force and maintain this force for 10 s. The CONTREX device marked the data. This procedure was repeated three times, with a rest period of 30 s in between trials. The results of each experimental test were not fed back to the participants.

For force sense data, the relative values of VE and AE were used for representation and the formulas are as follows:

$$\text{RAEFS}=\frac{\text{AE}}{25\text{\%MVIC}}$$ (3)

$$\text{RVEFS}=\frac{\text{VE}}{25\text{\%MVIC}}$$ (4)

Notes: RAEFS: relative absolute error of force sense; RVEFS: relative variable error of force sense.

Dynamic postural control-Dynamic postural control was evaluated using the Y-balance test (Move2Perform, Evansville, IN, United States), which was verified to have good interrater test-retest reliability (Overmoyer and Reiser., 2015). The participants stood barefoot at the centre joint of the standing board whilst their toe was pointed at the red line for maintaining the position of the foot. Their hands were placed on the hips during the test. They were instructed to reach as far as possible with the non-supporting legs in the anterior, posterolateral and posteromedial directions. The participants performed six trials to eliminate the learning effect before the testing procedure. When a participant had the following conditions, the trial must be retested: 1) fails to keep balance during the test; 2) the heel of the supporting leg is lifted off the ground and 3) unable to return to the initial position after reaching. The average reach distance was then normalised to leg length, which measured from the anterior superior iliac spine to medial malleolus.

### Static postural control

**FIGURE 2:**
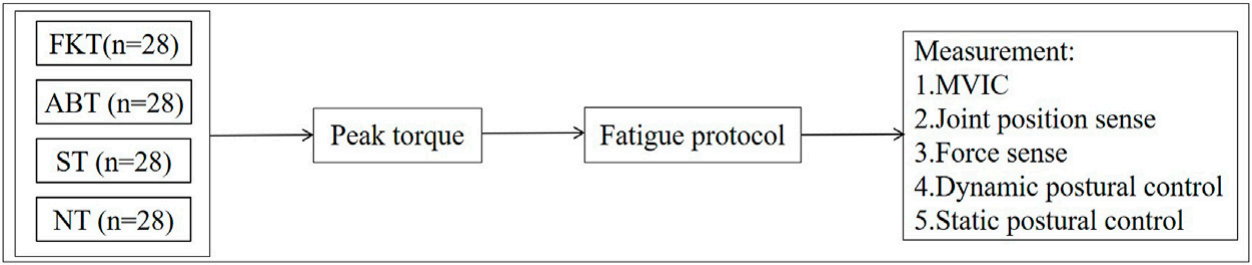
A flowchart of the experimental procedures.

For static postural control tasks, the participants were asked to keep standardised unilateral stance with barefoot on a force platform (KForce, KINVENT, United States) for 30 s. During the test, the participants stood on the test limb and kept their hands on their hips and their non-test limb maintained hip flexion at 30° and knee flexion at 45°. Subsequently, the tests were performed with the participants’ eyes closed. All values were collected at a sample rate of 75 Hz. The Center of pressure (COP) data generated from the force platform were analysed using the code written in Matlab (Mathworks Inc., Natik, MA), which included 1) mediolateral COP sway range (mm); 2) anteroposterior COP sway range (mm); 3) mean velocity of mediolateral COP sway (mm/s); 4) mean velocity of anteroposterior COP sway (mm/s) and 5) sway area (mm^2^), which is the elliptical area of the COP points. The above tests were performed in random order. Figure 2 presents a schematic flowchart of the entire procedure.

### Statistical analysis

Data were presented as mean and standard deviation (SD). The normality of distribution of data was tested using Shapiro–Wilk test. One-way repeated measures ANOVA was used in determining whether a significant difference exists amongst the taping conditions. Statistical significance was set at alpha <0.05. Bonferroni tests were conducted for post-hoc analysis when the effect of the test was significant. All data were analysed with SPSS 26 (IBM Corp., Armonk, NY, United States).

## Results

The results showed no significant difference in the four taping treatments in JPS and FS measurements. Similarly, no significant difference was found for the MVIC of plantarflexion and dorsiflexion in all four taping treatments (Table 1).

**TABLE 1:** Comparison of parameters in JPS, FS and MVIC among four taping treatments.

	NT	FKT	ABT	ST	F	P	η^2^
PF-AEJPS(°)	1.33 ± 0.77	1.33 ± 0.63	1.23 ± 0.69	1.30 ± 0.59	0.152	0.928	0.006
DF-AEJPS(°)	1.50 ± 0.91	1.31 ± 0.82	1.54 ± 0.86	1.40 ± 0.63	0.792	0.502	0.280
PF-VEJPS(°)	0.62 ± 0.29	0.50 ± 0.26	0.60 ± 0.44	0.51 ± 0.37	0.777	0.510	0.028
DF-VEJPS(°)	0.47 ± 0.29	0.53 ± 0.35	0.41 ± 0.20	0.57 ± 0.39	1.448	0.235	0.510
PF-RAEFS(°/N)	1.33 ± 0.77	1.33 ± 0.63	1.23 ± 0.69	1.30 ± 0.59	0.152	0.928	0.006
DF-RAEFS(°/N)	1.50 ± 0.91	1.31 ± 0.82	1.54 ± 0.86	1.40 ± 0.63	0.792	0.502	0.028
PF-RVEFS(°/N)	2.11 ± 1.43	2.02 ± 1.39	2.21 ± 1.42	1.96 ± 1.26	0.221	0.881	0.008
DF-RVEFS(°/N)	0.94 ± 0.67	0.83 ± 0.51	0.72 ± 0.58	2.60 ± 9.14	1.063	0.313	0.038
PF-MIVC(N)	83.04 ± 25.56	89.46 ± 30.29	87.71 ± 26.45	88.39 ± 31.68	1.672	0.179	0.058
DF-MVIC(N)	25.87 ± 8.21	24.86 ± 7.71	24.76 ± 7.51	25.68 ± 8.53	0.524	0.667	0.019

Note: Values are means ± standard deviation (SD); Significant differences (*p* < 0.05); PF, plantarflexion; DF, dorsiflexion; MVIC, maximum voluntary isometric contraction; AEJPS, absolute error of joint position sense; VEJPS, variable error of joint position sense; RAEFS, relative absolute error of force sense; RVEFS, relative variable error of force sense; NT, no taping; FKT, facilitatory kinesio taping; ABT, ankle balance taping; ST, sham taping.

**FIGURE 3:**
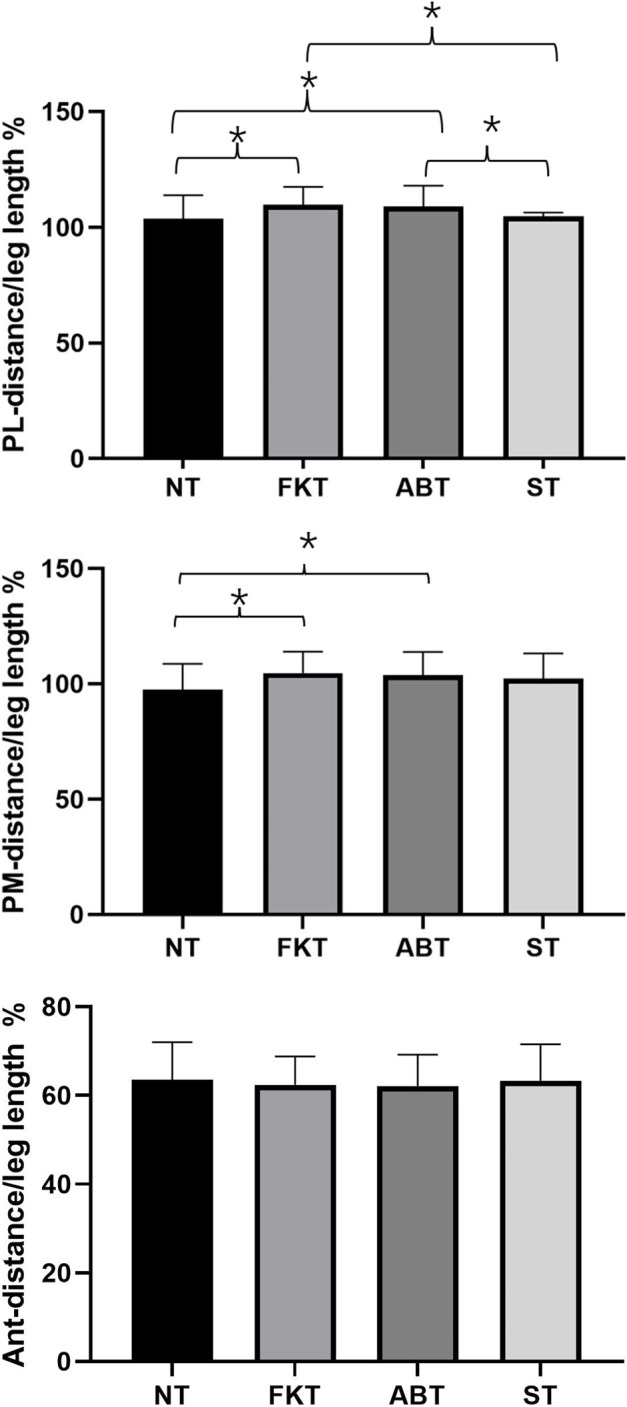
Comparison of parameters in Y-Balance scores amongst four taping treatments. *p < 0.05, Ant, anterior; PL, posterolateral; PM, posteromedial.

For Y-balance test, a significant difference was observed amongst the four taping treatments in posterolateral direction [F (3,81) = 11.9, *p* < 0.001, η^2^ = 0.291]. Post-hoc analysis showed that the mean of normalised reach distance of NT was significantly lower than that of FKT [*p* < 0.001, d = −6.099, 95% confidence interval (CI) = −9.839, −2.360%] and ABT (*p* < 0.001, d = −5.439, 95% CI = −8.941, −1.937%). The mean of normalised reach distance of ST was significantly lower than that of FKT (*p* < 0.001, d = −5.095, 95% CI = −7.777, −2.413%) and ABT (*p* < 0.001, d = −4.435, 95% CI = −7.075, −1.796%). Similarly, a significant difference was found amongst the four taping treatments in posteromedial direction [F (3,81) = 7.099, *p* < 0.001, η^2^ = 0.208]. The post-hoc analysis also showed that the mean of normalised reach distance of NT was significantly lower than that of FKT (*p* = 0.003, d = −7.1123, 95% CI = −12.242, −2.005%) and ABT (*p* < 0.001, d = −6.258, 95% CI = −10.138, −2.378%, Figure 3). For the anterior direction, no significant differences were observed in the four treatments (*p* = 0.751).

Regarding COP data, a significant difference was found amongst the four taping treatments for mediolateral COP sway range when eyes were closed. The post-hoc analysis showed that the mediolateral COP sway range of NT was significantly larger than that of FKT (*p* = 0.003, d = 3.750, 95% CI = 1.093–6.406 mm) and ST (*p* < 0.001, d = 3.551, 95% CI = 1.472–5.630 mm, Table 2).

**TABLE 2:** Comparison of parameters in COP for different conditions.

	NT	FKT	ABT	ST	F	P	η^2^
ML COP sway range (mm)	13.72 ± 6.22	9.97 ± 4.22	11.55 ± 5.31	10.17 ± 4.36	9.116	<0.001	0.252
AP COP sway range (mm)	12.22 ± 3.16	13.08 ± 5.41	13.14 ± 5.43	14.08 ± 6.14	1.041	0.379	0.037
ML COP velocity (mm/s)	8.53 ± 7.51	8.55 ± 9.89	8.61 ± 9.19	6.12 ± 7.46	0.657	0.545	0.024
AP COP velocity (mm/s)	7.75 ± 6.24	13.41 ± 18.60	11.26 ± 15.45	8.39 ± 6.60	1.153	0.323	0.41
Sway area (mm^2^)	114.92 ± 60.65	86.51 ± 69.30	101.22 ± 63.55	94.00 ± 50.00	1.465	0.23	0.051

Note: Values are means ± standard deviation (SD); Significant differences (*p* < 0.05); ML, mediolateral; COP, left of pressure; AP, anteroposterior; NT, no taping; FKT, facilitatory kinesio taping; ABT, ankle balance taping; ST, sham taping.

## Discussion

This study aimed to understand the effects of different KT methods on postural control after ankle muscle fatigue in individuals with FAI. The findings showed that KT caused a significant increase in the posterolateral and posteromedial reached distances of Y-balance test. In static postural control, proprioception and muscle strength findings demonstrated no differences amongst the four taping conditions.

Isokinetic fatigue protocols have been widely used to induce ankle muscle fatigue. Castillo et al. (2022) demonstrated that the fatigue of ankle muscle using isokinetic dynamometer was associated with deficits in postural control and reduced the functional performance. Similarly, Jahjah et al. (2018) used a similar protocol to cause fatigue in ankle muscles and observed worse joint position sense in the ankle. Impairment of neuromuscular control caused by fatigue severely affects dynamic joint stability and the body’s intrinsic protection from injury, particularly in participants with FAI. In addition, Yaggie and McGregor (2002) found that the changes in postural control were transient and recovered within 20 min. Therefore, the post-fatigue test in the current study was completed within 15 min after fatigue exercise.

To the authors’ knowledge, limited studies regarding the effect of KT in participants with FAI following ankle muscle fatigue are available. The current study found that KT has no effects on ankle proprioception or muscle strength in individuals with FAI after ankle muscle fatigue. In line with these results, Zanca et al. (2015). found no effect of KT application for shoulder proprioception following muscle fatigue in healthy individuals. Strutzenberger et al. (2016). also reported that KT did not influence lower limb strength in young adults compared with that in groups with no taping. The possible reason behind the ineffectiveness of KT may be that it could mediate the influence of fatigue on the ankle. However, the efficacy provided by KT may not be sufficiently strong to improve the performance in proprioception or muscle power.

The significant findings in Y-balance test are consistent with those in previous studies. Choi and Lee (2020) found that ABT could improve dynamic balance with eyes open and closed after ankle muscle fatigue in healthy individuals. Lin et al. (2020) found that KT provides a better postural control during landing following muscle fatigue than ankle brace in athletes with FAI. The significantly improved dynamic balance could be attributed to tactile stimulus and the mechanoreceptor stimulation supported by KT, compensating for the loss of the afferent feedback caused by fatigue. The current study also found that KT application significantly improved the dynamic balance but only in posterolateral and posteromedial directions. The reason may be that the different directions in the Y-balance require activation and contribution of different muscles. In the anterior direction, knee extensor and hip abductor are the most active muscles (Nelson et al., 2021) and the effects of KT are more localised to the area of its usage (Boozari et al., 2018), which may explain the significant results not present in the anterior direction. By contrast, Kodesh and Dar (2015) found that KT application did not contribute to Y-balance performance following ankle muscle fatigue in participants with CAI. And Esfandiari et al. (2020) demonstrated that KT did not affect the dynamic balance of young healthy women after local fatigue. The possible causes of the inconsistency may be the differences in KT application techniques.

In addition, according to the results of COP data, the mediolateral COP sway range of FKT and ST conditions improved compared with that of NT condition after ankle muscle fatigue. This finding may be due to the placebo effect of KT. de-la-Torre-Domingo et al. (2015) evaluated the immediate and prolonged effects of KT on balance in participants with CAI and found that the balance of the KT and control groups improved and that no difference was found between them. The authors concluded that the finding may be related to the increase in confidence after taping application. Mak et al. (2019) also found that the function of KT on muscle strength increase could be attributed to a placebo effect. Although the mechanism of the placebo effect of KT is unclear, it may be beneficial for preventing injury by contributing to stronger expectations and confidence.

Furthermore, no differences were found between the methods of KT application in regard to tests. These results are similar to those of previous studies investigating different KT applications. Fereydounnia et al. (2019) compared the effect of two methods of KT on muscle strength, balance and functional performance in participants with or without FAI and found no differences between the methods of application. Previous studies also concluded that the effect of KT is independent of the application direction (Choi and Lee, 2019). These results suggested that the effect of KT may not be related to the methods of application. However, Yu et al. (2021) argued that only long length of KT improved ankle inversion proprioception for healthy individuals. One explanation for these conflicting findings is that the long length of KT was across both the ankle and knee joints, it is possible that participants may have acquired more proprioceptive information.

Some limitations should be acknowledged in this study. Firstly, the participants could not be blinded to the methods of taping. Thus, the psychological effects of KT could not be ruled out. Secondly, some of the recruited participants were specialised in one sport and their performance in the tests may be inevitably affected to some degree by their skills. Thirdly, this study only indicated the acute effect of KT. Future studies should investigate the long-term effects of KT.

## Conclusion

The current study demonstrated that KT increased dynamic postural control in individuals with FAI after ankle muscle fatigue, and this effect is not strongly related to the taping methods. By preventing fatigue-related impairments of postural control, KT may help reduce the risk of injury in individuals with FAI.

## Data Availability

The original contributions presented in the study are included in the article/supplementary material, further inquiries can be directed to the corresponding author.
